# Ecological drivers of evolution of swine influenza in the United States: a review

**DOI:** 10.1080/22221751.2025.2455598

**Published:** 2025-01-16

**Authors:** Varun Goel, Jessica Ding, Bijaya Hatuwal, Emily Giri, Thomas J. Deliberto, James Lowe, Richard Webby, Michael Emch, Xiu-Feng Wan

**Affiliations:** a Department of Geography, University of South Carolina, Columbia, SC, USA; b Department of Geography and Environment, University of North Carolina, Chapel Hill, NC, USA; c NexGen Center for Influenza and Emerging Infectious Diseases, University of Missouri, Columbia, MO, USA; d Department of Electrical Engineering & Computer Science, College of Engineering, University of Missouri, Columbia, MO, USA; e Christopher S. Bond Life Sciences Center, University of Missouri, Columbia, MO, USA; f US Department of Agriculture Animal and Plant Health Inspection Service, Fort Collins, CO, USA; g Department of Veterinary Clinical Medicine, University of Illinois at Urbana-Champaign, Urbana, IL, USA; h Department of Infectious Diseases, St. Jude Children's Research Hospital, Memphis, TN, USA; i Department of Epidemiology, University of North Carolina, Chapel Hill, NC, USA; j Department of Molecular Microbiology and Immunology, School of Medicine, University of Missouri, Columbia, MO, USA

**Keywords:** Influenza A virus, swine Influenza virus, H1N1, H3N2, ecology, commercial swine farm, evolution

## Abstract

Influenza A viruses (IAVs) pose a major public health threat due to their wide host range and pandemic potential. Pigs have been proposed as “mixing vessels” for avian, swine, and human IAVs, significantly contributing to influenza ecology. In the United States, IAVs are enzootic in commercial swine farming operations, with numerous genetic and antigenic IAV variants having emerged in the past two decades. However, the dynamics of intensive swine farming systems and their interactions with ecological factors influencing IAV evolution have not been systematically analysed. This review examines the evolution of swine IAVs in commercial farms, highlighting the role of multilevel ecological factors. A total of 61 articles published after 2000 were reviewed, with most studies conducted after 2009 in Midwestern US, followed by Southeast and South-central US. The findings reveal that ecological factors at multiple spatial scales, such as regional transportation networks, interconnectedness of swine operations, farm environments, and presence of high-density, low-genetic diversity herds, can facilitate virus transmission and enhance virus evolution. Additionally, interactions at various interfaces, such as between commercial swine and feral swine, humans, or wild birds contribute to the increase in genetic diversity of swine IAVs. The review underscores the need for comprehensive studies and improved data collection to better understand the ecological dynamics influencing swine IAV evolution. This understanding is crucial for mitigating disease burden in swine production and reducing the risk of zoonotic influenza outbreaks.

## Introduction

Influenza A viruses (IAVs) are responsible for substantial human morbidity and mortality and continue to present a substantial public health challenge. In addition to the impact of annual seasonal outbreaks, IAVs are concerning due to their pandemic potential. There have been four documented influenza pandemics in recent history – the 1918 H1N1, 1957 H2N2, 1968 H3N2, and 2009 H1N1 pandemics. These pandemics occurred when a novel IAV evolved with surface glycoproteins—hemagglutinin (HA) and neuraminidase (NA)—that were antigenically distinct from those previously circulating in human populations [1].

Wild aquatic birds act as the major reservoir for the large genetic diversity of IAVs, facilitating the emergence of new viruses that can infect humans, other mammals, and domestic poultry. Pigs, harbouring both avian-like and human-like virus receptors in their respiratory epithelial cells, have been proposed as “mixing vessels” for pandemic IAV strains by fostering genetic reassortment [2]. This genetic reassortment between avian, swine, and human IAVs occurs when pigs are co-infected by two or more distinct IAV strains, leading to the formation of novel viruses with potential pandemic implications [3]. The 2009 H1N1 pandemic is an example, originating from a reassortment between North American triple-reassortant swine H3N2 and Eurasian avian-like swine H1N1 viruses [4]. In response, the United States (US) formalized a national surveillance system to monitor swine herds for novel IAVs, highlighting the critical role of ongoing surveillance due to pigs’ susceptibility to diverse IAV strains [5].

Swine production is currently the fastest growing livestock sector, consisting of intensive systems in which hundreds or thousands of pigs are farmed in high-density facilities [6]. Within these intensive livestock production systems, pigs can act as significant intermediate and amplifying hosts for zoonotic viruses with pandemic potential [7]. Highly industrialized swine farming operations feature low genetic diversity and high densities in swine herds, both of which facilitate viral transmission and evolution [8]. While intensive animal farming operations are typically considered to operate as “closed systems”, large-scale swine production facilities have structural vulnerabilities that can compromise biosecurity, increasing the risk of disease transmission among animals and raising concerns about the potential of zoonotic diseases such as influenza [9].

**Figure 1.**
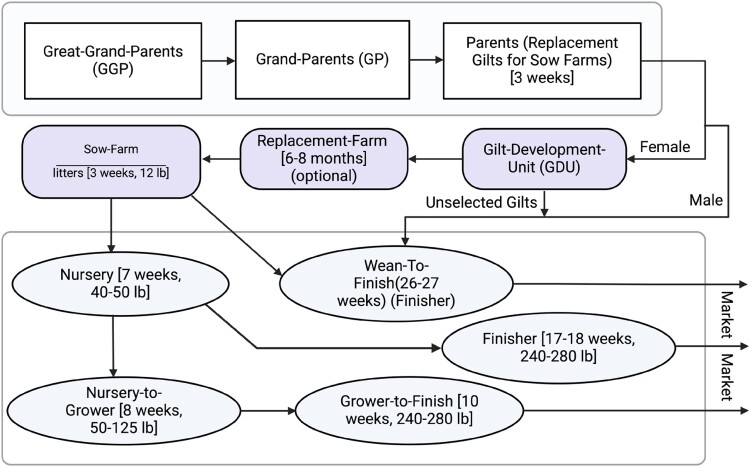
A typical commercial swine operation infrastructure. The operation begins with Great-Grand-Parents (GGP), genetically selected pigs that produce Grand-Parents (GP), who in turn breed parent pigs destined to replenish the population in Sow-Farms (SF). Male pigs are usually sent directly from weaning to Wean-to-Finish (WF) farms, while females are transferred to Gilt-Development-Units (GDU). The most suitable gilts from GDUs are then moved to SF, although some systems may place them in a replacement farm before their final transfer to SF. The farming practices vary across different systems, but the typical pathways post-farrowing at SF include: (i) from Nursery to Finisher farm, (ii) from Nursery to Nursery-to-Grower and then to Grower-to-Finish farm, and (iii) directly from SF to Wean-to-Finish (WF) farm. The figure was generated using Biorender and adapted from Hatuwal et al. [14].

The contemporary intensive swine farming operations consist of various types of farms based on the weight and stages of pig growth (Figure 1). These operations typically include Great-Grand-Parents (GGP) farms, Grand-Parents (GP) farms, Parent farms, Gilt-Development Units (GDU), Replacement Farms, Sow Farms, Nursery, Nursery-to-Grower, Grower-to-Finish units, Wean-to-Finish units, and Finisher units, although the specific composition may vary from system to system. These farming sites can be situated within the same region or across different regions, or within the same state or spanning multiple states. Consequently, pigs may need to be relocated frequently between these farming sites. About 80% of swine in North America and Europe, and approximately 50% in Asia, are housed in intensive farming systems [10,11]. In China, swine are often raised in backyard settings, typically in smaller numbers and with significantly lower levels of biosecurity compared to intensive farming operations [12]. The genetic diversity of pigs in backyard settings is generally much larger compared to those in commercial settings [13].

Overall, North America and Europe are regions with a high risk of IAV transmission and spillover, mainly attributed to swine movement, including both domestic transport and international exports [15]. In contrast, in Asia there is a high risk for reassortment between avian and swine IAVs, primarily due to close proximity of pigs, poultry, and wild birds, with lower biosecurity measures within their swine farming system [16]. However, in many cases, influenza infections in pigs result in mild clinical signs with relatively low mortality rates, leading to the perception within the swine industry that influenza poses minor economic risk. As a result, these infections are often overlooked, enabling the virus to become enzootic and to persistently evolve within these high-density production systems [17].

Although surveillance and documentation of swine IAV evolution is extensive, the ecological factors driving this evolution remain poorly understood. This review describes the ecological determinants influencing swine IAV evolution, focusing on environmental factors, management strategies, and their impact across different spatial scales. Understanding the ecological dynamics at the farm, system, and regional levels is necessary to enhance management strategies and surveillance to mitigate the disease burden in swine production and lower the risk of future pandemics. The focus of this review is on the ecological factors influencing the dynamics of swine IAV evolution in swine production systems in the US. The US plays an important role in global swine production, and is the second-largest producer, seventh-largest importer, and second-largest exporter of pork globally [18]. This paper supplements existing swine IAV reviews in the literature, including those that provide general descriptions of swine IAV in the US [19–21] and those that focus specifically on swine production in the US [22].

## Methods

### Search strategy

The preparation of this review followed the PRISMA-Scr guidelines [23]. We searched for peer-reviewed articles on PubMed and Web of Science using terms and keywords related to swine, influenza type A, and US (Supplement 1). We only included articles published in or after the year 2000 to capture the introduction of the predominant co-circulating swine IAVs.

### Article selection and inclusion criteria

We only considered articles that were written in English and had full text available. Articles were considered only if they reported studies that either collected primary data or used secondary data on swine IAV obtained from swine production systems in the US. While our primary focus was on commercial swine in the US, we also included studies involving exhibition swine, feral swine, and the interface between swine and humans, particularly those providing data for comparison with swine IAVs in commercial swine. Likewise, we also considered IAV data from other countries on a case-by-case basis, especially if the study included analysis of data on commercial swine in the US. Additionally, given the emphasis of this review on the ecology and evolution of swine IAVs, we prioritized studies that analysed data related to virus evolution (i.e. genetic sequences) rather than those only on epidemiological data. Finally, we included studies that analysed data on at least one ecological factor that may be related to swine IAV evolution. While ecological factors can be broadly defined, we considered factors at multiple spatial scales including regional-, system-, farm-, and herd-level factors related to commercial swine production systems in the US. Additionally, we also included factors linked to commercial swine interactions with other important hosts in the ecosystem such as humans, feral swine, and avian hosts.

### Categorization of ecological factors

Commercial swine operations in the US operate across a range of spatial scales, including operations that may span across multiple geographic regions and states. Hence, in addition to the specific herd-level practices and individual sites where swine herds are kept, other ecological factors such as the movement of herds across different farming sites and regions can also influence swine IAV evolution in the US. To better highlight the role of ecological factors relevant to commercial swine production, we categorize these factors into nested spatial scales: (1) region-level factors that operate across states, (2) system-level factors that operate at the scale of a coordinated commercial swine operation (which may or may not span multiple states), (3) farm-level factors that are specifically linked to an individual farming site housing one or more swine herds, and (4) herd-level factors such as age or vaccination status that are generally specific to each swine herd within farms.

In addition to these nested factors specifically associated with commercial swine operations, there are other ecological factors that influence IAV transmission and evolution but operate at scales beyond those of commercial swine operations. These factors are linked to conditions that may increase contact between commercial swine and other IAV hosts such as humans, feral swine, exhibition swine, wild birds, and domestic poultry. In this review, we categorize these factors at the scale of the “landscape” which refers to the scale at which hosts, environment, and pathogens interact together. These landscape-level factors highlight the documented characteristics of human-swine, domestic swine-wildlife, and swine-avian interfaces relevant to swine IAV transmission and evolution.

## Results

### Descriptive statistics

**Figure 2.**
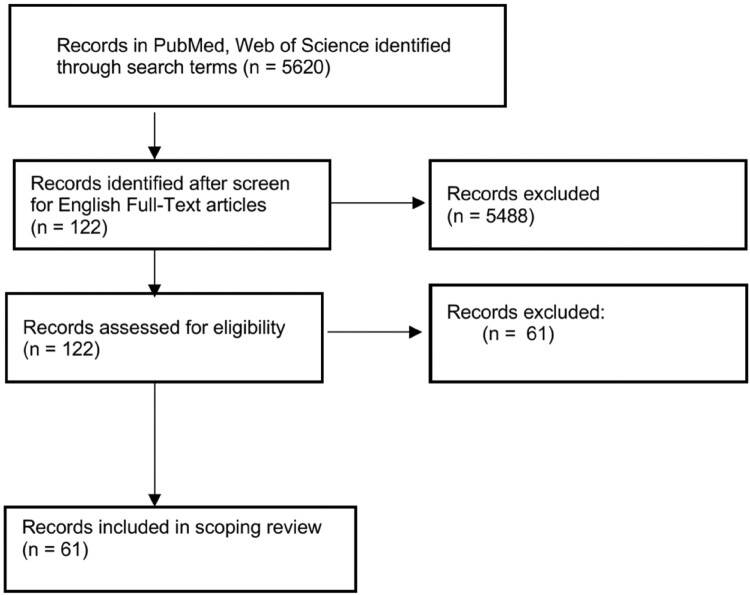
Flow diagram for article selection: out of 122 screened articles, 61 articles were considered eligible for inclusion into the scoping review. In addition to duplicates, other 61 records were excluded on a case-by-case basis.

Of 5,620 articles screened in the initial database search, 122 articles were selected for manual screening. After screening for duplicates and selecting relevant articles summarizing the ecology and evolution of swine IAVs, 61 were included in the final review (Figure 2). As found in reviews related to swine IAVs, most papers were published after the 2009 H1N1 pandemic (n = 54) (Table 1) [8,24]. While some studies either undertook a country wide analysis, or analysis within a single state, most studies spanned multiple states, such as those in the Midwest, followed by South-central and Southeastern states. Among all articles on commercial swine (n = 46), 26 were published from studies at the national, regional, or county level, followed by articles that contained data at the local level such as farms and swine herds (n = 20). Additionally, five articles measured interactions between commercial swine and other hosts such as humans, feral swine, exhibition swine, wild birds, or domestic poultry. Around half of the articles (n = 31) were published using primary data sources, with the remaining articles (n = 30) using secondary data sources. Four articles contained results from both primary and secondary data.

**Table 1.** Distribution of records by year, spatial extent, host, resolution, and data type for the articles (n = 61) reviewed in this study. For spatial resolution, only articles focusing on commercial swine (n = 46) were considered.

Variables	Categories	Total (N = 61)
**Year Published**	Before/in 2009	7
	After 2009	54
** **		
**Spatial Extent**	Midwestern US	40
	Southeast/South-central US	23
	Countrywide	8
** **		
**Primary Host Type**	Commercial swine	46
	Cross species	5
	Others	10
** **		
**Spatial Resolution (n = 46)**	Regional	15
	System level	11
	Farm level	15
	Herd/pig level	5
** **		
**Data Type**	Primary	31
	Secondary	30
	Both	4

### Summary of evolution of swine IAV in the US

IAVs present in the domestic swine populations of the US are genetically and antigenically diverse, and the evolution of these viruses have persisted over the last century, particularly during the last three decades. During the past decade, the most common circulating strains in domestic swine were IAV subtypes H1N1, H1N2, and H3N2. These subtypes are enzootic in domestic swine populations and further form at least 22 genetic clades or subclades of H1 and 24 clades and subclades of H3 (Supplement figure S1). Additionally, occasional spillovers of various other IAV subtypes, including H4N6, H2N3, and H3N1 from avian species, and H1 and H3 IAVs from humans, were reported. However, most of these spillovers were transient and resulted in only limited spread. While the main focus of this review is on ecological factors influencing swine influenza evolution, we briefly summarize major evolutionary events in the attached supplement.

We also note that in the literature, multiple nomenclatures have been used to characterize the genetic and antigenic diversity of enzootic H1 and H3 swine IAVs, including the alpha-beta based system for H1 and H3, and the recent clade/subclade system for both. To facilitate correlation with existing studies, we have listed the corresponding lineages reported in the literature, with a primary focus on those found in US swine farms (Supplement Table S1) [25].

### Ecological factors linked to swine IAV evolution among commercial swine

#### Regional factors

Analyses of data from multiple years of the US Department of Agriculture (USDA) passive surveillance systems suggests that while the major swine IAV subtypes – H1N1, H1N2, and H3N2 – are consistently detected across the US, genetic clades and lineages varied regionally [26]. Regional clustering of genetic diversity of swine IAVs in US swine populations is especially prominent in the Midwest and North Carolina, the two places where commercial swine operations are most heavily clustered. In the Southeastern states of North Carolina and South Carolina, IAV strains from the γ and δ lineages are the most prevalent, while in the Midwest, classical α and β lineages are more common [27]. In the NA lineage, viruses containing N2.1998A and N2.2002A are geographically limited to the Midwest, primarily in Iowa or adjacent states, while viruses with N2.1998B are more frequently detected in southeastern states including North Carolina [28].

While there are distinct patterns of genetic diversity based on region, swine farms in the Midwest harbour the greatest potential for viral reassortment due to the region’s higher swine densities and its commercial role as a final marketing destination and net importer of swine [29]. Midwestern states – Iowa, Missouri, Illinois, Indiana, and Ohio – are the nation’s largest producers of corn and soybeans used to feed livestock including pigs, and represent the nation’s largest market for feeder pigs, particularly those from North Carolina and Oklahoma [30]. Swine in North Carolina and Oklahoma are considered the key “source” populations for human origin H1N1 and H1N2 respectively, and the rapid diffusion of these viruses to the Midwest through commercial swine transportation “swineways” suggests that the Midwestern US may serve as an ecological “sink” for these viruses [35]. Similarly, phylogenetic analysis has shown that N2 clades N2.1998B and N2.2002B moved from North Carolina to the Midwest potentially due to frequent interstate movement, with the Midwestern states of Indiana, Iowa, and Illinois further acting as sources of N2.2002B dissemination to other nearby states [28]. Notably, there is sparse evidence of IAV dissemination in the opposite direction from midwestern states to southeastern and south-central states such as North Carolina and Oklahoma, or between the southeastern and south-central states. Additionally, these “swineways” may also include long-distance transportation routes between Canadian provinces such as Manitoba and Ontario, and midwestern states such as Iowa, Minnesota and Illinois. The movement of pigs across these inter-country networks may be attributed to the introduction of the H1α−3 clade into US swine herds, that has now spread rapidly into other parts of western and eastern US [31].

Due to the directional flow of pigs into the Midwestern US, commercial swine herds within these states contain the greatest genetic diversity of IAV in the US [32]. The Midwest represents not only the greatest number of unique genomic constellation patterns, but also the largest abundance of collected genome sets of swine IAV in the US. Furthermore, several studies show that the majority of Midwestern farms have multiple genetically diverse IAV viruses co-circulating, which can further facilitate reassortment events between different strains [29,33,34]. It is important to note however, that while most H1 clades tend to spread in the Midwest through outside transportation networks, H1γ may be an exception. H1γ, which was first identified in Iowa in 2003, was later detected in Texas before spreading to the Midwest and becoming endemic in North American swine populations by 2004 [35]. This could potentially be attributed to the transportation of exhibition swine, which make up approximately 1.5% of the total US swine population but experience different transportation and population dynamics compared to commercial swine [36].

#### Operation associated factors

***System-level factors****:* Large industrial swine farming operations that specialize in a single growth-phase of production have largely replaced farrow-to-finish operations that performed all growth-phases of swine production from birth to slaughter. Each of these operations at a farming site are part of coordinated swine production systems specifically responsible for one growth-stage of the production purpose (Figure 1). This multi-site swine production system that is prevalent in the US establishes interconnected swine transportation networks between farming sites, particularly those within the same system. These networks exhibit a “small world” structure, in which a minority of farms are highly interconnected and account for the majority of animal movements [30]. These highly interconnected farms act as hubs for both incoming and outgoing pig flows, putting them at higher risk of both receiving and spreading infections [30,37]. Additionally, long-distance swine transportation networks associated with large-scale commercial swine production may facilitate the rapid spread of diseases across geographic areas. This phenomenon contributes to the observed similarities in certain IAV clades over large areas where IAV genetic diversity is otherwise distinct [26].

Such interconnectedness exposes commercial swine farms to IAV infections from multiple sources and is shown to facilitate emergence of novel swine IAV reassortants [38]. Individual single growth-phase production sites, such as GDUs, nurseries, and sow farms, which typically have a higher prevalence of IAV [39], serve as sites of IAV introduction and dissemination to other farms within the same system or even across multiple systems from the swine transportation networks [40]. Early growth phase farms in the production cycle appear to be particularly susceptible to IAV infection. This susceptibility can be attributed to several factors, such as a low level of influenza specific herd immunity, frequent internal transportation of pigs from external sources, and the presence of a large number of immunologically naive hosts. Among these early growth phase farms, nurseries may act as sources of IAV transmission due to frequent out-transportation of feeder pigs whereas finishing farms are at a higher risk of becoming sinks for IAV transmission due to in-transportation [41]. This was supported by a prior study demonstrating that that finisher herds were only IAV-positive if the sow herds from which they sourced their swine were IAV-positive [42]. In addition, finishing farms can serve as risk sites for the emergence of novel IAV strains, with additional exposures from the transportation network [41].

***Farm-level Factors***: In addition to the specific growth-phase of production assigned to a farm, various factors including husbandry practices, herd housing, environmental conditions, and biosecurity measures, may influence IAV transmission and genetic diversity of IAVs. For example, practices such as all-in all-out (AIAO) management, in which groups of animals closely matched by age, weight, or production stage are moved together from one production stage to the next, are associated with lower odds of IAV positivity among nurseries and finisher farms, compared to farms that practice continuous flow or AIAO-by-room [39,42].

Similarly, conditions associated with housing of swine herds that may increase mixing and contact between pigs such as large herd sizes, farm-level and pen-level pig density, and proximity to other barns are also associated with higher IAV prevalence, which may also subsequently increase genetic diversity of IAV [24]. In addition to housing conditions of pigs, indoor environmental conditions associated with transitioning from open-ventilation to closed-ventilation systems during the onset of cold weather are also associated with increased IAV positivity [5]. In addition to colder temperatures, the reduced air exchange and decreased relative humidity may increase the likelihood of swine IAV outbreaks, similar to what has been observed for human influenza outbreaks [39,43]. Despite its unclear effects on pig-barn environmental conditions, windspeed could be important when analysed along with downwind or upwind direction from a nearby farm as it could result in IAV exposure [39].

Farms across all stages of production also differ in biosecurity practices that may affect the risk of IAV introduction and dissemination. However, even with the use of personal protective equipment (PPE) and other hygiene measures, IAV transmission may occur through fomites. IAV RNA can persist on boots and coveralls after contact with infected pigs. This is important as such modes of fomite transmission may persist despite changing PPE and taking other protective measures [44].

***Herd and Pig-level Factors***: While farm-level practices may explain variation in rates of IAV prevalence and genetic diversity between farms, high levels of genetic diversity may also exist within a farm. Multiple studies have demonstrated co-circulation of different HA and NA subtypes and their variants in piglets prior to weaning, both within farms and even within individual piglets. There may be viral reassortments at the farm level, as evident by the presence of multiple genotypes within a single farm, with differences observed in only a single gene segment in some cases [34].

Such genetic diversity of IAVs within individual piglets in the same farm may be partially explained by immune selection pressure due to influenza vaccination and presence of maternally-derived antibodies. In particular, piglets (pigs less than 21 days of age) were more likely to test positive for IAV compared to those more than 4 weeks old [45]. This may partly be explained by diverse levels of maternally-derived antibodies in piglets, which can provide varying degrees of protection against virus infections. Additionally, these antibodies may hinder pigs from developing active immunity to IAV vaccination. Due to the high antigenic drift among swine IAV viruses, vaccines may have limited success in reducing IAV genetic diversity or evolutionary rates among herds, but may also lower viral replications which reduces the likelihood of viral reassortment [46]. In a study comparing boosted pigs with unvaccinated pigs, it was found that vaccinated pigs had more reassortant viruses and distinct genotypes compared to vaccinated pigs, but within-pig nucleotide variations, nucleotide polymorphisms, and evolutionary rates were similar for both H1N1 and H3N2 viruses regardless of vaccination status [46]. In another study, it was found that pigs with higher maternally-derived antibody titres in a nursery farm had been shown to be at higher risk of recurrent infections, especially for a heterologous strain due to interference with pigs’ humoral response [47].

#### Landscape-level factors and other IAV hosts

Given the potential for pigs to act as “mixing vessels”, there are concerns that interactions between commercial pigs and other IAV hosts such as feral swine, exhibition swine, wild birds, poultry, and humans may give rise to novel reassortants with pandemic potential. We summarize the empirical evidence of important factors driving swine IAV evolution at interfaces with other IAV hosts.

***Domestic-feral swine interface:*** The US is home to more than 5 million feral swine spread across at least 35 states [48]. Unlike their domestic counterparts, feral swine have unrestricted movement, leading to unique encounters with wildlife and livestock (including domestic swine) in diverse habitats. Genetic reassortment and the emergence of novel IAV strains have been observed in domestic-feral swine interactions in the southeastern and south-central US. Feral swine are predominantly exposed to H1 and H3 subtypes, which are genetically similar to enzootic domestic swine IAVs, indicating spillover from domestic to feral swine [49,50]. In a study analysing serum samples from feral swine across 35 states between 2010 and 2013, it was found that among the IAV-positive samples, more than half showed cross-reactivity with swine IAVs, 1 with avian IAVs, and some with both avian and swine IAVs [51]. This suggests that feral swine had been exposed to both swine and avian IAVs, with a higher prevalence of exposure to swine IAVs. Generally, contact between feral swine and domestic swine is more likely in states with smaller farms, such as backyard or farrow-to-finish farms that have less stringent biosecurity measures. Despite concerns, there is no evidence of IAV transmission from feral swine back to commercial swine although this has not been studied rigorously.

***Domestic swine – avian interface:*** Wild waterbirds such as waterfowl (e.g. ducks, geese, and swans), shorebirds, and gulls are the primary natural reservoirs of IAVs [52], and the majority of these birds are migratory birds which may share habitats on their migratory pathways [53]. Sporadic and independent introductions of various subtypes of avian IAVs from wild birds to domestic poultry, including H3, H4, H5, H6, H7, H9, and H10, have been documented in Canada, South Korea, China, Indonesia, the US, and Mexico [54]. While the majority of other AIV spillover events have been documented in East and Southeast Asia, there have been sporadic cases of avian IAV spillover into swine in North America. The spillover of subtype H4N6 avian IAVs to swine was detected in North America in Ontario, Canada in 1999 and caused limited transmission in the swine population [55]. Another independent introduction of subtype H4N6 was reported in Missouri in 2016, with an additional four suspected cases in the index breeding and gestation farm [56]. There were also reports of the detection of avian H3N3 in 2001 and avian H1N1 in 2002 in Canadian swine [55,57], and of two avian H5N2 in 2016 in Guanajuato in Mexico [58,59]. However, all of these avian IAVs detected in swine were quickly eliminated, and none of them led to outbreaks or contributed to the genetic diversity of swine IAVs in the US.

***Domestic swine – human interface:*** Documented IAV transmission from pigs to people has resulted in human pandemics such as the 2009 pandemic, and *vice-versa* in pig enzootics or even panzootics [60]. Although swine IAV spillover from swine to humans generates greater public health interest, sequence data suggests that humans contribute more to genetic diversity of IAVs in swine than swine to those in humans [61]. A pandemic IAV can rapidly spread worldwide within months and potentially within days with increasing globalization, and dramatically increase opportunities for pigs to be exposed to viruses through humans. Once a pandemic occurs, these pandemic viruses are transmitted quickly back to pigs across continents and rapidly become panzootic in pigs, enriching the IAV genetic pool in pigs, enhancing IAV evolution at the human-swine interface, and facilitating the emergence of novel enzootic and pandemic IAVs. For example, the 1918 H1N1 pandemic virus became the common ancestor of the classical swine H1N1 IAV, and contributed to genetic segments in three recent pandemic viruses and all seasonal IAVs in humans [62]. The 1957 H2N2 pandemic IAV likely emerged from H2 avian IAV and H1N1 human IAV [1]; the 1968 H3N2 pandemic IAV likely emerged from H3 avian IAV and the H2N2 human IAV [1], and the 2009 H1N1 pandemic virus [A(H1N1)pdm09] had PB2 and PA genes from avian IAVs, the PB1 gene from human H3N2 IAV, and other genes from swine IAVs [63]. After all pandemic outbreaks, these viruses were soon detected in swine populations across Asia, Europe, and North America.

Since 2009, the rapid spread of A(H1N1)pdm09 within swine populations in the US and worldwide, has significantly diversified the IAV genetic pool within the swine population. In North America alone, within a single year, at least 9 genetic reassortants derived from A(H1N1)pdm09 were detected in the domestic swine population [64,65], and a H3N2 variant (H3N2v), which has a matrix gene of A(H1N1)pdm09, was frequently detected in both domestic swine [66] feral swine [49]. While some studies have evaluated the ecology linked to transmission of swine IAVs from pigs to humans, primarily in children in agricultural fairs [67], ecological factors linked to human IAV transmission in swine are far more limited, especially for the US.

In the context of commercial swine, farmworkers are an important population as they may not only test positive for IAVs but also potentially carry them to other communities. Specific sites such as breeding herds that tend to be IAV reservoirs and have deeper involvement of farmworkers with pig handling as compared to other sites are important sites for potential IAV evolution [68]. Similarly, farmworkers that vaccinate piglets are more likely to be contaminated and may infect susceptible piglets resulting in circulation of IAV. Due to the difficulty associated with enrolling swine farm workers in the US, there is limited evidence comparing farm worker and swine samples from the same farm. However, in one study enrolling swine workers at farms in Minnesota, it was found that there were workers that had swine-origin IAV strains in their nasal passages, and at least one worker was infected with human seasonal-H1N1 IAV of pandemic origin [69].

## Knowledge gaps

This review summarizes the documented ecological factors affecting the transmission and evolution of swine IAV in the US. Overall, we found that, while there are several studies on risk factors for IAV transmission in commercial swine, empirical evidence that quantifies how ecological factors drive swine IAV evolution is limited. Below, we summarize key limitations and knowledge gaps.

### Data availability

Most studies, especially those that use secondary data rely on the USDA swine influenza surveillance database. Despite being extensive, the sequences submitted as part of the database often lack detailed metadata on farm location, pig flow, vaccination status, and other relevant information. Because the data relies on anonymous submissions and is not harmonized, other important information on variables such as herd size, farm type, or herd age is often missing. Additionally, most data is either collected during high transmission seasons or during outbreak events and may not reflect the overall prevalence of IAV strains. Similarly, the data is based on opportunistic sampling, and it tends to over-represent larger commercial swine farming systems and operations. This may lead to biased results if data is not treated properly with robust techniques such as downsampling and bootstrap analysis.

### Landscape-level analysis

While there is a better understanding of how regional to local factors related to commercial swine farming systems may influence swine IAV evolution, empirical evidence on the role of landscape factors, especially those that may be associated with cross-species transmission in the US is very limited. There is a relative lack of research examining the environmental and ecological variables associated with swine IAV and its interactions with feral hogs, wild birds and poultry. The data on human interaction and swine IAV evolution is also sparse. This is partly due to the challenges associated with acquiring data on feral hogs, wild birds, and human populations such as farm workers.

However, with increasingly available fine-scale spatiotemporal environmental data to measure habitats, remotely-sensed products to estimate swine farm locations, and temperature and precipitation products, models can be used to create landscape-level surfaces at these cross-species interfaces. For example, a study assessing the potential of contact between swine backyard farms and feral hogs combined various remotely-sensed products with maximum entropy (MaxEnt) models to estimate feral hog habitats and their seasonality [70]. As feral hog populations increase across the US and in areas with larger commercial swine farms, and novel highly pathogenic avian IAV strains that may more efficiently evolve in mammals are discovered, the interface between domestic swine, feral, hogs, wild birds, and humans becomes even more important.

### Linking swine IAV ecology and evolution

This review shows that there are major gaps in our understanding of swine IAV ecology and in particular, the ecological factors that may promote or hinder IAV evolution. Currently, several studies evaluate the role of risk factors on swine IAV exposure and positivity but not on virus evolution. Understanding the evolutionary dynamics of IAV is crucial for assessing its potential to cause pandemics and developing effective control strategies.

Whole-genome sequencing (WGS) plays a critical role in identifying reassortments and novel subtypes. However, the majority of studies have relied on serological tests, which only provide insights into historical exposure and lack the resolution needed to understand the genetic diversity of viruses in the swine population. Therefore, they cannot accurately reflect viral evolution or provide information to assess viruses with pandemic potential. Incorporating WGS techniques, particularly second and third-generation sequencing platforms like Illumina, Roche 454 GS Junior, Ion Torrent PGM, and Nanopore MinION, enables the identification of quasi species and mixed infection. These advances can significantly enhance our understanding of IAV evolution beyond HA and NA subtypes of the viruses.

Additionally, the emerging interdisciplinary field of landscape genetics combines theories and methods from population genetics and landscape ecology to explore spatial variation in genetics. By exploring interactions between evolutionary mechanisms and environmental and ecological features, we can assess gene flows across heterogenous landscapes and fine spatial, temporal, and molecular scales that may drive infectious disease emergence and spread [71]. This approach has previously been applied to evaluate the landscape drivers of H5N1 avian influenza molecular change in Vietnam [72]. A key part of this approach is its incorporation of both population and environmental drivers at multiple spatial and temporal scales in understanding what factors promote or prevent gene flow and potential for reassortment. Given the complicated ecology of swine IAV, the need to understand both local and regional drivers, and its potential for cross-species transmission, a landscape genetics approach may help us better understand the ecological and evolutionary pressures of swine IAV evolution. By addressing these gaps, we can increase our understanding of IAV transmission dynamics and evolutionary processes, and facilitate the development of effective control strategies to mitigate the risk of IAV outbreaks and pandemics.

## Conclusion

In summary, several studies have extensively documented the substantial antigenic and genetic diversity of swine IAVs in the US, which exhibit significant regional variation. Specifically, the Midwest plays a critical role in the dispersion of IAVs due to its position as a key commercial hub for domestic swine herds, receiving large numbers of pigs from both the southern states and Canada. This has resulted in the Midwest harbouring the highest antigenic and genetic diversity of swine IAVs in the country. The combination of high swine densities and large genetic diversity of IAVs circulated in this region creates a conducive environment for the genetic reassortment among IAVs, leading to the emergence of new virus strains.

Despite evidence of multiple IAV subtypes co-circulating in the Midwest and instances of genetic reassortment at the farm level, the broader implications of these dynamics for the long-term evolution and global persistence of novel IAV strains remain unclear. Moreover, there is a lack of understanding regarding the specific local and regional factors that drive viral evolution. Current studies often use generalized state-centred geographic models and are limited by the lack of detailed metadata, a consequence of the sensitive nature of disease surveillance and the anonymity of data submissions. Additionally, there is a notable gap in research examining how specific farm-level factors – such as indoor environments, farm types, vaccination practices, and biosecurity measures – impact the evolution of IAVs in commercial swine. Research on the potential for virus transmission and reassortment between commercial and feral swine populations, as well as between swine and avian species, is also sparse. This highlights a critical need for more detailed and comprehensive studies to understand the complex dynamics of IAV evolution and spread within and beyond the US.

## Supplementary Material

TableS1_final.xlsx

figureS1.jpg

SIAV_review_supplement.docx
