# Supplementary figures and images for: Ecological drivers of evolution of swine influenza in the United States: a review
Source: Emerg Microbes Infect. 2025 Jan 16;14(1):2455598. doi: 10.1080/22221751.2025.2455598 (PMC11780704; doi:10.1080/22221751.2025.2455598)

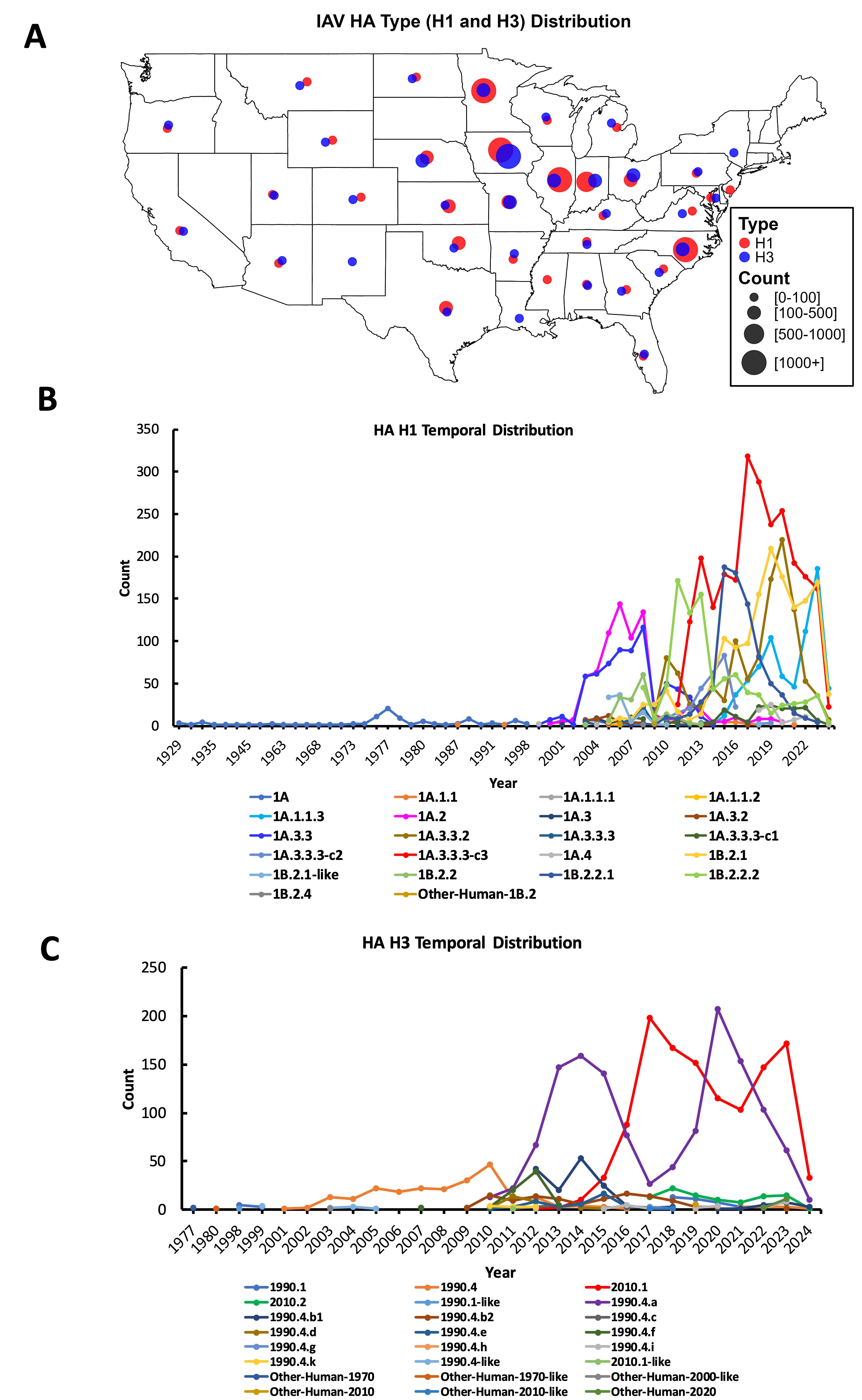

Supplement: figureS1.jpg [file TEMI_A_2455598_SM8476.jpg]
