# Supplementary material for: Ecological drivers of evolution of swine influenza in the United States: a review
Source: Emerg Microbes Infect. 2025 Jan 16;14(1):2455598. doi: 10.1080/22221751.2025.2455598 (PMC11780704; doi:10.1080/22221751.2025.2455598)
Supplement: SIAV_review_supplement.docx [file TEMI_A_2455598_SM8475.docx]

**Supplement**

**1 Search terms for review**

The following search terms were used in Pubmed and Web of Science to screen articles related Swine Influenza in the United States

| **PubMed** |  |
| --- | --- |
| (((swine[Title] OR pig*[Title] OR piglet*[Title] OR gilt*[Title] OR boar*[Title] OR sow*[Title] OR weaner*[Title] OR hog*[Title] OR porcine[Title] OR pork*[Title] OR "Sus scrofa"[Title] OR "Sus domesticus"[Title] OR "suidae"[Title] OR livestock[Title] OR herd*[Title] OR farm*[Title] OR flock*[Title] OR mammal*[Title] OR animal*[Title] OR zoo*[Title]) NOT ("guinea pig"[Title] OR "guinea pigs"[Title])) AND (influenza OR flu))  AND (United States OR USA OR US) OR (("Swine*"[MeSH]) AND ("Influenza A virus*"[MeSH]))  [AND ("2000/01/01"[Date - Publication] : "3000"[Date - Publication])] | |
| **Web of Science** |  |
| TITLE:(( swine OR pig* OR piglet* OR gilt* OR boar* OR sow* OR weaner* OR hog* OR porcine OR pork* OR "Sus scrofa" OR "Sus domesticus" OR "suidae" OR livestock OR herd* OR farm* OR flock* OR mammal* OR animal* OR zoo*) NOT ("guinea pig" OR "guinea pigs"))  AND TOPIC:(influenza OR flu)  AND TOPIC:(United States OR USA OR US) (note: no "All fields" nor "keywords" only, "topic" relates to title, abstract and keywords)  [Year: from 2000 to 2024] | |

**2** **Linking common swine IAV nomenclatures in literature**

[Insert Table S1 here]

**Table S1:** Table linking previous designations of H1 and H3 clades in the US with their global nomenclature. Adapted from <https://influenza.cvm.iastate.edu/clades.php>. with permission of authors.

**2 Evolution of swine IAV in the United States**

**3 Evolution of swine IAV in the United States**

*3.1 Genetic and antigenic drift*

In the US, the H1N1, including multiple lineages stemming from the 1918 H1N1 pandemic, and H3N2, which originate from human seasonal H3N2 influenza viruses along with their derived HA-NA reassortant subtypes, are the main IAV subtypes identified in swine populations. In comparison, in Eurasia, in addition to these H1N1 and H3N2 lineages, avian-like H1 IAVs have also been co-circulating in domestic swine population since the 1970s [1].

In U.S. swine populations, multiple co-circulating genetic clades and subclades have been reported, many of which are antigenically distinct [2–5]. As of April 2024, GISAID has archived whole or partial genomic sequences of 11,289 subtype H1 and 4,489 subtype H3 swine IAVs collected from U.S. swine populations. Based on the HA gene-based global clade naming definition [6], these contemporary viruses include at least 22 subclades of H1 and 24 clades/subclades of H3 viruses (Figure S1). These variants were identified across 38 states, primarily in the Midwest and Southern U.S., aligning with the density of swine populations. Of these variants, 1A.3.3.3-c2 is the most common clade/subclade for H1 swine IAV, and 2010.1 and 1990.4.a for H3 swine IAV. The majority of these genetic variants were detected after the year 2000, covering the time period of this review.

**Figure S1**: Temporal and geospatial distribution of swine IAV HA (H1 and H3) types in the US till 2024 April from GISAID data. A) geospatial distribution of H1 and H3 clade/subclades in the US. B) Temporal distribution of H1 clade/subclades in the US. C) Temporal distribution of H3 clade/subclades in the US. The contemporary H1 swine IAVs in the US can be classified into at least 22 clades/subclades, including 1B.2.2.1, 1A.3.3.3-c1, 1A.1.1.3, 1A.3.3.3-c3, 1B.2.1, 1A.3.3.2, 1B.2.2.2, 1A.2, 1A.3.3, 1A.3.3.3-c2, 1B.2.2, 1A.4, 1A, 1A.3.2, 1A.1.1, 1A.3, 1A.3.3.3, 1B.2.1-like, Other-Human-1B.2, 1A.1.1.1, 1A.1.1.2, and 1B.2.4; the contemporary H3 swine IAVs in the US can be classified into 24 clades/subclades, including 2010.1, 1990.4.a, 1990.4.b1, 1990.4.b2, 1990.4, Other-Human-2010, 1990.1, 2010.2, 1990.4.k, 1990.4.e, 1990.4.i, 1990.4.d, 1990.4.c, 1990.4.f, 1990.4.g, 1990.1-like, Other-Human-2000-like, 1990.4-like, Other-Human-1970, Other-Human-2020, Other-Human-2010-like, 1990.4.h, 2010.1-like, and Other-Human-1970-like.

Among the neuraminidase (NA) genes, N1 and N2 subtypes circulate within the US, with N2 subtype detected more frequently in 65% of reported swine IAVs [7,8]. Within N1, there are two genetic clades; the N1.Classical clade emerged with the introduction of the 1918 H1N1 into swine [9] while the N1.Pandemic Eurasian swine lineage gene emerged following the 2009 H1N1 human pandemic [10,11]. Within N2, there are primary two circulating genetic clades, both associated with human to swine transmission events; N2.1998 was introduced into swine in the late 1990s with a triple reassortant human seasonal IAV [12], while N2.2002 was introduced in early 2000s during a human to swine spillover event associated with H1 Delta clades [13]. Another N2 lineage was detected in 2016 but is infrequently detected [14], as opposed to N2.2002 which is the most common, and N2.1998, which is also sustainably detected [15]. Since their introductions into swine, the two N2 lineages have undergone significant antigenic drift and have expanded their genetic diversity, with two distinct monophyletic clades within each lineage [16]. Additionally, it is observed that rates of evolution among N2 clades and H1 and H3 clades are similar, and that there is preferential pairing among specific NA and HA genetic clades across swine populations in the US [17].

Compared to those in HA and NA, genetic variants in the internal genes are much less characterized, despite reports of widespread genetic mutations, including amino acid substitutions, in swine IAVs [18,19].

*3.2 Genetic reassortants*

In addition to variants arising from accumulating mutations, genetic reassortants are frequently detected in swine populations, contributing significantly to genetic diversity in U.S. swine populations. These reassortants have been reported not only within H1 or H3 subtypes of swine IAVs but also between H1 and H3 subtypes, and even with human-origin IAVs that have spilled over from humans [12,20,21].

Among all these reassortants, the H3N2 triple reassortant virus detected in 1998 was perhaps the most influential genetic variant, significantly predominating in the genetic pools of swine populations, rapidly increasing the genetic diversity of swine IAVs, and facilitating the emergence of the 2009 H1N1 pandemic virus in humans [22]. The H3N2 triple reassortant viruses contain gene segments from classical swine H1N1 IAV (NP, M, and NS), human seasonal H3N2 IAV (PB1, HA, and NA), and avian IAV (PB2 and PA) [20]. These triple-reassortant viruses in swine went on to reassort with human H3N2 viruses and co-circulating classical H1N1, with reassortment events mainly involving the HA and NA segments [23]. These reassortant viruses further reassorted with gene segments from Eurasian swine viruses, contributing to the genetic constellation of the 2009 H1N1 pandemic virus [1] . It is believed to have emerged in swine populations in Mexico before spreading to humans [24]. Studies also indicate that the virus may have circulated undetected in swine populations for 8-10 years before its emergence in humans [10,25].

The inter-HA subtype reassortants detected in swine populations include H3N1 and H1N2, with H1N2 being one of the major reassortants reported [3]. Interestingly, the reassortments among these genetic pools were not random; certain genetic variants of HA are more likely to reassort with other heterosubtypes. However, unliked those used for HA, there is still a lack of standardized nomenclature for genetic reassortants. Thus, there has been no systematic analysis of reassortants, making it difficult to align reassortant events consistently due to variations in identification protocols across individual reports.

**References**

[1] Zhu H, Zhou B, Fan X, et al. Novel Reassortment of Eurasian Avian-Like and Pandemic/2009 Influenza Viruses in Swine: Infectious Potential for Humans. J Virol. 2011;85(20):10432–10439.

[2] Kitikoon P, Nelson MI, Killian ML, et al. Genotype patterns of contemporary reassorted H3N2 virus in US swine. J Gen Virol. 2013;94(6):1236–1241.

[3] Martin BE, Sun H, Carrel M, et al. Feral Swine in the United States Have Been Exposed to both Avian and Swine Influenza A Viruses. Appl Environ Microbiol. 2017;83(19):e01346-17.

[4] Rajao et al. Antigenic and genetic evolution of contemporary swine H1 influenza viruses in the United States. 2018;518:45–54.

[5] Feng Z, Baroch JA, Long L-P, et al. Influenza A Subtype H3 Viruses in Feral Swine, United States, 2011–2012. Emerg Infect Dis. 2014;20(5):843–846.

[6] Anderson TK, Macken CA, Lewis NS, et al. A Phylogeny-Based Global Nomenclature System and Automated Annotation Tool for H1 Hemagglutinin Genes from Swine Influenza A Viruses. mSphere. 2016;1(6):e00275-16.

[7] Anderson TK, Nelson MI, Kitikoon P, et al. Population dynamics of cocirculating swine influenza A viruses in the United States from 2009 to 2012. Influenza Other Respir Viruses. 2013;7:42–51.

[8] Zeller MA, Anderson TK, Walia RW, et al. ISU FLUture: a veterinary diagnostic laboratory web-based platform to monitor the temporal genetic patterns of Influenza A virus in swine. BMC Bioinformatics. 2018;19(1):397.

[9] Koen J. A practical method for field diagnosis of swine disease. Am J Vet Med. 1919;14:468–470.

[10] Smith GJD, Vijaykrishna D, Bahl J, et al. Origins and evolutionary genomics of the 2009 swine-origin H1N1 influenza A epidemic. Nature. 2009;459(7250):1122–1125.

[11] Dawood F, Novel Swine-Origin Influenza A (H1N1) Virus Investigation Team. Emergence of a Novel Swine-Origin Influenza A (H1N1) Virus in Humans. N Engl J Med. 2009;360(25):2605–2615.

[12] Zhou NN, Senne DA, Landgraf JS, et al. Genetic Reassortment of Avian, Swine, and Human Influenza A Viruses in American Pigs. J Virol. 1999;73(10):8851–8856.

[13] Vincent AL, Ma W, Lager KM, et al. Characterization of a newly emerged genetic cluster of H1N1 and H1N2 swine influenza virus in the United States. Virus Genes. 2009;39(2):176–185.

[14] Zeller MA, Li G, Harmon KM, et al. Complete Genome Sequences of Two Novel Human-Like H3N2 Influenza A Viruses, A/swine/Oklahoma/65980/2017 (H3N2) and A/Swine/Oklahoma/65260/2017 (H3N2), Detected in Swine in the United States. Matthijnssens J, editor. Microbiol Resour Announc. 2018;7(20):e01203-18.

[15] Walia RR, Anderson TK, Vincent AL. Regional patterns of genetic diversity in swine influenza A viruses in the United States from 2010 to 2016. Influenza Other Respir Viruses. 2019;13(3):262–273.

[16] Kaplan BS, Anderson TK, Chang J, et al. Evolution and Antigenic Advancement of N2 Neuraminidase of Swine Influenza A Viruses Circulating in the United States following Two Separate Introductions from Human Seasonal Viruses. J Virol. 2021;95(20):e0063221.

[17] Zeller MA, Chang J, Vincent AL, et al. Spatial and temporal coevolution of N2 neuraminidase and H1 and H3 hemagglutinin genes of influenza A virus in US swine. Virus Evol. 2021;7(2):veab090.

[18] Mehle A, Doudna JA. Adaptive strategies of the influenza virus polymerase for replication in humans. Proc Natl Acad Sci. 2009;106(50):21312–21316.

[19] Aguirre VMC, Mercado-García M del C, Trujillo-Ortega ME, et al. Genetic changes detected in the internal genes of porcine influenza viruses isolated in Mexico. Vet México OA [Internet]. 2014 [cited 2024 Aug 19];1(1).

[20] Webby RJ, Swenson SL, Krauss SL, et al. Evolution of Swine H3N2 Influenza Viruses in the United States. J Virol. 2000;74(18):8243–8251.

[21] Khiabanian H, Trifonov V, Rabadan R. Reassortment Patterns in Swine Influenza Viruses. Stolovitzky G, editor. PLoS ONE. 2009;4(10):e7366.

[22] Garten RJ, Davis CT, Russell CA, et al. Antigenic and Genetic Characteristics of Swine-Origin 2009 A(H1N1) Influenza Viruses Circulating in Humans. Science. 2009;325(5937):197–201.

[23] Nelson MI, Lemey P, Tan Y, et al. Spatial Dynamics of Human-Origin H1 Influenza A Virus in North American Swine. PLOS Pathog. 2011;7(6):e1002077.

[24] Mena I, Nelson MI, Quezada-Monroy F, et al. Origins of the 2009 H1N1 influenza pandemic in swine in Mexico. eLife. 2016;5:e16777.

[25] Nelson MI, Stratton J, Killian ML, et al. Continual Reintroduction of Human Pandemic H1N1 Influenza A Viruses into Swine in the United States, 2009 to 2014. J Virol. 2015;89(12):6218–6226.
